# High-Strength and Conductive Electrospun Nanofiber Yarns

**DOI:** 10.3390/polym16223137

**Published:** 2024-11-11

**Authors:** Qingqing Shao, Bo Xing, Zhaoqun Du, Weidong Yu

**Affiliations:** 1 Key Laboratory of Textile Science and Technology (Ministry of Education), College of Textiles, Donghua University, Shanghai 201620, Chinabo.xing@ensait.fr (B.X.); 2 Department of Macromolecular Chemistry II and Bavarian Polymer Institute, University of Bayreuth, 95440 Bayreuth, Germany

**Keywords:** electrospinning, high strength, nanofiber yarn, sliver nanowires, electrical property

## Abstract

In electrospinning, nanofibers are frequently produced in nonwoven web form. Their poor mechanical properties (below 100 MPa) and difficulty in tailoring the fibrous structure have restricted their applications. However, advanced materials must be highly resistant to both deformation and fracture. By combining electrospinning technology with stretching, we have overcome this disadvantage and demonstrated a polyacrylonitrile nanofiber yarn with a tensile strength of 743 ± 20 MPa. The nearly perfect uniaxial orientation of the fibrils under the stretching process is crucial for the remarkable mechanical properties of the yarn. Additionally, the nanofiber yarn was functionalized by a dip-coating process with silver nanowires (AgNWs), imparting conductive properties. This conductive, high-strength nanofiber yarn demonstrates practical applications in flexible and wearable devices. The presented strategy is versatile and can be adapted to create other high-performance nanofiber yarns, with potential uses in fields such as biomedicine and smart textiles.

## 1. Introduction

Nanofibers have attracted considerable attention due to their ultrafine dimensions and extensive surface area-to-volume ratio, making them invaluable in various applications, including filtration [1,2], biomedicine [3,4], and energy storage [5,6]. It is widely acknowledged that electrospinning is the preferred technique for fabricating nanofibers due to its straightforward and adaptable methodology, which enables the production of fibers with a diameter ranging from nanometers to micrometers [7]. Electrospun nanofibers have many applications, including tissue engineering [8,9,10], sensors [11], filtration [12,13] and catalysis [14].

Despite the wide applicability of electrospun nanofibers, most existing electrospun fibers are produced in the form of randomly oriented nonwoven mats [15,16]. This random fiber arrangement, coupled with their nonwoven structure, results in relatively low mechanical strength and limits the effective transfer of load along the fiber axes [17]. As a result, these materials often lack the structural integrity required for high-performance applications [18,19,20,21]. While some studies have attempted to address these issues by developing electrospun nanofiber yarns, their mechanical properties and functional capabilities remain constrained due to insufficient fiber alignment and structural cohesion [22,23,24,25,26,27]. Additionally, the integration of conductive elements into electrospun nanofiber yarns has proven challenging, with limited approaches to achieving reliable, uniform conductivity without sacrificing mechanical properties.

To address these limitations, this study introduces a novel approach that combines electrospinning with a controlled hot drawing process to produce polyacrylonitrile (PAN) nanofiber yarns with enhanced mechanical properties. PAN, known for its semi-crystalline structure, is particularly suitable for electrospinning due to its stability and potential for high mechanical performance. The hot drawing process aligns the fibrils uniaxially, improving crystallinity and molecular orientation, which are essential for high-strength and high-modulus yarns. Moreover, we impart conductivity to these high-strength nanofiber yarns through a dip-coating process with silver nanowires (AgNWs), creating a conductive network on the nanofiber surface. This dual enhancement of mechanical strength and electrical conductivity enables the nanofiber yarns to function in advanced applications, such as wearable electronic textiles and implantable devices.

The remainder of this paper is organized as follows: Section 2 details the materials and methods used, including the electrospinning, drawing, and dip-coating processes. Section 3 presents the results and discussion, focusing on the structural, mechanical, and electrical properties of the PAN nanofiber yarns. Section 4 provides the conclusions, summarizing the main findings and potential applications of this novel high-performance nanofiber yarn.

## 2. Materials and Methods

**Materials:** Ethylene glycol (EG, 99.8%, VWR), silver nitrate (AgNO3, ≥98%, VWR), N, N’-dimethylformamide (DMF, 99.99%, Fisher Chemical, Waltham, MA, USA), Eisen(Ⅲ)-chlorid (FeCl3, 99% Grussing), Poly(vinyl pyrrolidone) (PVP, number average molar mass (Mn) of 1,300,000, Aldrich, St. Louis, MO, USA), Polyethylenimine (PEI, branched, Mn of 10,000, Aldrich), Polyacrylonitrile (PAN, Mn of 120,000, Dolan, London, UK), acetone and ethanol (technical grade) were used in this study. AgNWs were synthesized by mixing all reaction agents and reacting at the desired temperatures in the famous polyol process [28].

**The preparation of high-strength nanofiber yarn:** Nanofiber yarns were obtained by yarn electrospinning [21]. A solution of 15% weight was made by dissolving 1 g of PAN powder in 4.75 g of DMF solution with 0.976 g of acetone. This solution was loaded into syringes with metal needles and connected to a DC power supply at +14.7 kV and −13.8 kV to create charged fibers. These fibers were directed towards a rotating funnel, forming a fiber membrane. The membrane was then drawn by a pre-suspended yarn into a winder collector at 15 rpm, creating a rotating fiber cone. Helical fibers from the cone were spiraled up, forming a continuous and twisted polymer yarn. The process was conducted under an infrared lamp at approximately 45 °C. All as-spun yarns were stretched at high temperatures of 130 °C and 160 °C using a fiber stretching device [26]. The stretch ratio is determined by the difference in speed between the front and back rollers: $SR\;\lambda =\frac{V_{f}}{V_{s}}$, where $V_{f}\;$ and $V_{s}\;$ represent the speed of fast roller and slow roller.

**Fabrication of conductive composite yarn:** The composite nanofiber yarns were manufactured by a dip-coating process. First, the nanofiber yarn was cleaned in ethanol for about 10 min and dried in air. The nanofiber yarn was then dipped into 1% PEI solution for 24 h and dried in a vacuum oven at 20 °C for 12 h. The nanofiber yarn was dipped into the AgNW aqueous suspension at room temperature and then dried at 80 °C for 12 h. This dip-coating process was repeated over 3 cycles to adjust the amount of AgNW coated on the yarn.

**Characterization:** Scanning electron microscopy (SEM) (Zeiss LEO1530, Bayreuth, Germany) was employed for observing PAN nanofiber yarns and AgNW networks. Thermogravimetric analysis (TG 209 F1 Libra, Bayreuth, Germany) was employed to characterize the properties of the yarns. The curves were obtained at a heating rate of 10 K/min under a nitrogen atmosphere. Tensile tests were performed using a tensile tester (zwickiLine Z0.5, BT1-FR0.5TN.D14, Zwick/Roell, Bayreuth, Germany) with a clamping length of 10 mm, a crosshead rate of 5 mm/min at 25 °C and a pre-tension of 0.005 cN. The load cell was a Zwick/Roell KAF TC with a nominal load of 20 N. For fatigue tests at maximum stress of 200 MPa for 500 cycles, the sequential tensile cycles were conducted by the above tensile test. The absorption spectrum of the solution was recorded by ultraviolet–visible (Jasco V-630, JASCO, Bayreuth, Germany) spectroscopy with an ECTS-761 spectrophotometer, using 10 mm path length quartz cuvettes. Wide-angle X-ray scattering measurements were performed with a Bruker D8 ADVANCE anode X-ray generator in Germany, set to 40 kV and 40 mA, utilizing Cu-Kα radiation of wavelength 0.154 nm. Before analysis, the yarns were organized into a bundle on the stage. The patterns were captured across a 2θ angle range of 8° to 36°, scanning at a rate of 0.05°/min, at a temperature of 25 °C. The electrical properties of the composite yarns were investigated by applying a constant voltage of 3 V by a Potentiostat/Galvanostat Machine (Interface 1010, Bayreuth, Germany) to record the current passing through the nanofiber yarns.

## 3. Result and Discussion

**Figure 1:**
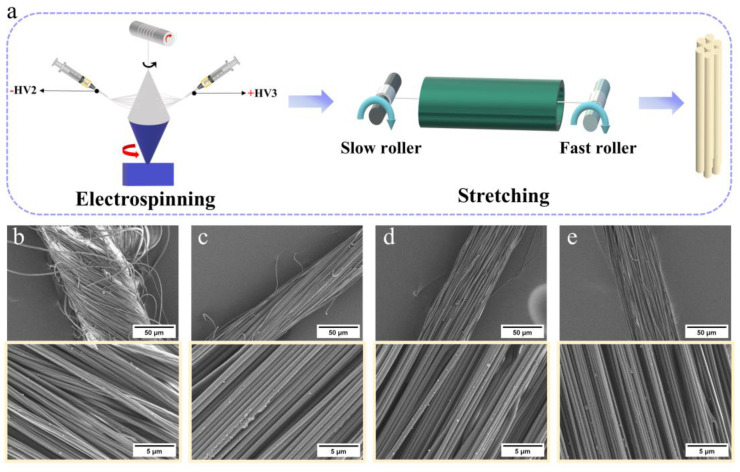
Preparation route (**a**). SEM images of different SRs; (**b**) SR 0, (**c**) SR 3, (**d**) SR 6 and (**e**) SR 8. SEM images with higher magnification are shown in the yellow frame.

High-strength nanofiber yarns were prepared as illustrated in Figure 1. First, a spinning solution with a concentration of 15 wt% was loaded into a syringe equipped with a metal needle to generate charged fibers under the influence of a high voltage. These fibers were then deposited into a rotating funnel, which slowly drew them into a coiled collector. During this process, nanofiber yarns were formed (Figure 1a). High-strength nanofiber yarns were obtained by stretching the nanofiber yarn at temperatures of 130–160 °C. The purpose of thermal drafting is to allow the fibers in the yarn to slip and align along the yarn. At the same time, stretching above the glass transition temperature can change the crystallinity of the yarn, which helps to improve the strength of the yarn. The high-strength nanofiber yarns were labeled SR x, where x represents the ratio of stretch. As the stretch ratio increases, the number of bent fibers in the yarn decreases significantly, the fibers gradually straighten (Figure 1b–e), and the fibers tend to be oriented along the axial direction of the yarn (in the yellow frame).

**Figure 2:**
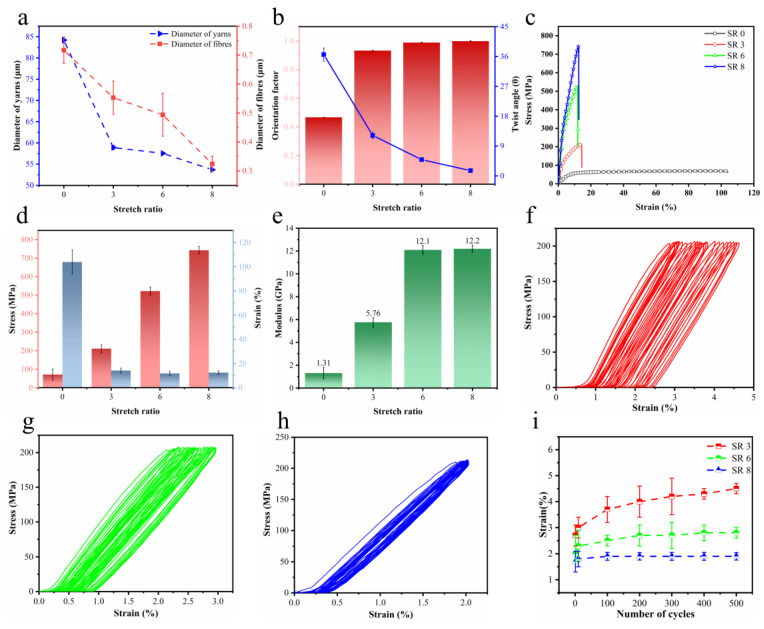
(**a**) Diameter of the yarns and nanofibers at SR 0, SR 3, SR 6, and SR 8. (**b**) Alignment factor and twist angle of yarns at different SRs. (**c**) Mechanical properties, (**d**) tensile strength and (**e**) modulus of yarns at different SRs. Stress/strain curves of yarns at (**f**) SR 3, (**g**) SR 6, and (**h**) SR 8 under maximum stress of 200 MPa for 500 cycles. (**i**) Changes in strain with different SRs over 500 cycles.

The Image J software (v1.54j) as used to process the SEM of the yarn (Figure 1) to obtain the diameter and twist angle of the yarns and fibers. As the stretch ratio increases, the diameter of the fibers in the yarn decreases (Figure 2a, red). This is due to the combined action of external tensile force and friction between fibers, whereby the curved fibers in the yarn are stretched and arrayed along the axial direction of the yarn. Yarn is defined as an aggregate composed of fibers; therefore, as the stretching ratio increases, the diameter of the nanofiber yarn also decreases (Figure 2a, blue). The Hermans orientation factor [29] is used to describe the degree of fiber alignment along the yarn axis.

(1) $$f=\frac{1}{2}\left(3\cos\theta^{2}-1\right)$$

In the formula, θ represents the angle between the fiber and yarn axes, which is equivalent to the twist angle. As the stretching ratio increases, the twist angle of the yarn gradually decreases, while the orientation degree increases significantly (Figure 2b, blue). As the draw ratio increases, the external force on the yarn also increases, which causes the fiber to be stretched along the axial direction of the yarn. This results in a decrease in the twist angle of the yarn and an increase in the orientation factor.

To analyze the effect of stretch on the mechanical properties of the yarns, the stress, strain and modulus were analyzed for nanofiber yarns with different SRs. Such stress–strain curves can vary greatly between the same type of nanofiber yarns from different SRs (Figure 2c). The variability is due to differences in stretch, once again emphasizing the importance of stretching during spinning and subsequent processing to control the mechanical properties of the yarn [30]. The maximum stress (Figure 2d) and modulus (Figure 2e) increased with the SR, whereas the elongation at break decreased with the SR. SR8 yarns have a tensile strength of 743 ± 20 MPa and a modulus of 12.2 ± 0.31 GPa, which is 10 times higher than the strength (70.8 ± 30 MPa) and modulus (1.31 ± 0.5 GPa) of unstretched yarn. The performance of SR8 yarn is similar to that of dragline silk [31,32].

The hysteresis of the cyclic stretching curve decreases as the stretching ratio increases, which indicates that the stretched yarn has higher stability (Figure 2f–h). Even after 500 cycles of loading and unloading at a maximum tensile strength of 200 MPa, only a negligible change in the final tensile strength (~1%) and deformation (~1.9%) was observed at SR8 (Figure 2i). SEM images confirmed that the heat stretching procedure altered the orientation of the nanofibers along the yarn’s main axis (Figure 1), with the percentage of aligned yarns increasing from 0.467 at no-stretch to 0.999 at SR 8 (Figure 2b). In addition, the diameter of the yarn decreased from 84.211 μm without stretching to 53.694 μm at SR 8. According to Peirce’s Theory [33], the smaller the yarn, the lower the probability of weak links. Consequently, the strength of the nanofiber yarn is directly proportional to its diameter.

**Figure 3:**
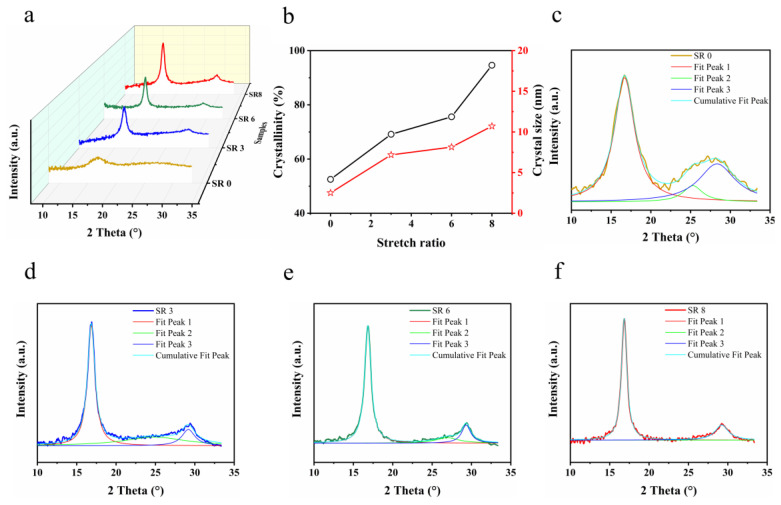
(**a**) WAXS spectra of yarns with different stretch ratios. (**b**) Dependence of the degree of crystallinity and crystallite size of yarns (corresponding to Figure 3a) as a function of the SR. The fits of yarns at (**c**) SR 0, (**d**) SR 3, (**e**) SR 6, and (**f**) SR 8.

Previous research has indicated that high crystallinity resulting from stretching is also a factor affecting the mechanical properties of yarns [30]. The WAXS spectra of yarns under different stretch conditions are depicted in Figure 3. All yarns exhibit a dominant and sharp diffraction peak around 2θ ≈ 16.8°, corresponding to the (100) plane in the hexagonal lattice of PAN. A weaker diffraction peak, attributed to the (101) plane of the hexagonal lattice, is discerned at approximately 2θ ≈ 29.0° [34,35] (Figure 3a). These observations substantiate that the PAN polymer is semi-crystalline. Notably, with an escalation in the stretch ratio, the relative intensity of the peak around 2θ ≈ 16.8° amplifies, implying an enhanced intermolecular interaction between the nitrile groups within the PAN macromolecular chains. However, the primary peak positions remain invariant, confirming that the stretching process does not disrupt the crystalline structure of PAN. Figure 3b displays the crystallinity and crystal size of the PAN (100) plane, deduced from Figure 3a. To determine the crystallinity and crystal size of samples, the peaks was first fitted with a Lorenz-peak function to obtain the area of separated peaks, which are depicted in Figure 3c–f. The crystallite size of the lateral-order domains was estimated by the Scherrer equation [36] as follows:

(2) $$L_{c}=\frac{K\lambda }{B\cos\theta }$$

where *λ* is the wavelength of the CuK_α_ X-ray, *B* is the full width at half maximum intensity (FWHM) of the peak around 2*θ* = 16.8°, and *K* is a constant of 0.89.

The crystallinity (CI) was determined by the Bell and Dumbleton method [36]:

(3) $$\mathrm{CI}=\frac{A_{c}}{\left(A_{c}+A_{a}\right)}$$

where *A*_c_ is the integral area of the crystalline zone around 2*θ* = 16.8° (fit peak 1) and 2*θ* = 29.1° (fit peak 3) in XRD patterns, and *A*_a_ is the integral area of the amorphous zone (fit peak 2) [37].

The crystallinity of PAN increases from approximately 52.5% (in the unstretched state) to roughly 94.6% (at SR 8). Concurrently, the crystal size experiences an increment during the stretching process, evolving from approximately 2.5 nm to 10.7 nm. This underscores that stretching prominently enhances both the crystallinity and crystal size. The stretching results in the macromolecular chains in the nanofibers being more likely to be arranged parallel to the axis, with a reduction in the distance and a closer packing of the chains [30,38]. This allows the formation of hydrogen bonds and regular arrangement of the molecules, thereby increasing the crystallinity of the fiber. In addition, the greater the degree of crystallinity and the size of the crystal particles, the more regular the arrangement of macromolecules in the yarn, the smaller the gap holes, the stronger the bonding between macromolecules and the base protofibril [39], and the greater the fiber’s breaking strength and initial modulus, but the greater the decrease in elongation (Figure 2).

**Figure 4:**
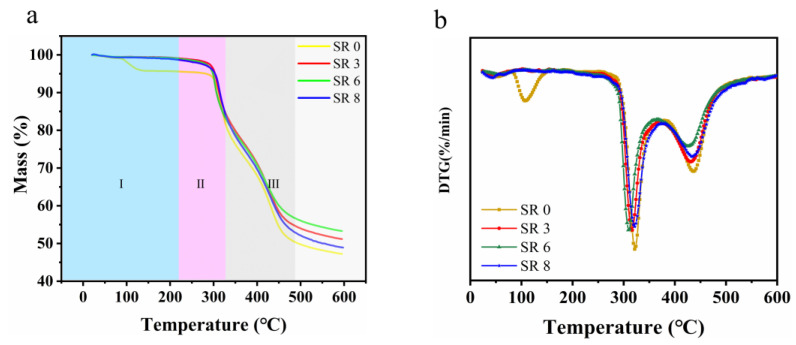
Thermogravimetric analysis (TGA) curves (**a**,**b**) derivative thermogravimetric (DTG) curves of samples in a nitrogen atmosphere.

The TGA and DTG curves, obtained at a heating rate of 10 K/min under a nitrogen atmosphere, are presented in Figure 4. Based on weight loss across different temperature ranges, three distinct phases can be identified in Figure 4a. TGA parameters, such as the initial decomposition temperature and the residual weight, are listed in Table 1.

In Phase I, the temperature ascends to around 250 °C with a minimal weight reduction observed [40]. Interestingly, the weight loss for the unstretched curve initiates earlier and leaves a smaller residual weight (95.43%) compared to the stretched yarns. This phenomenon is attributed to the fluffier structure of the unstretched yarns, housing a larger number of water molecules and low-molecular-weight oligomers. Within this phase, the nitrile groups in PAN undergo cyclization reactions, forming some ladder-like structures.

Phase II sees the temperature rise to about 350 °C, during which a broad and rapid weight loss occurs. This can likely be ascribed to a substantial random scission of the linear PAN chains, which do not partake in cyclization reactions during the heat treatment. Concurrently, the residual mass of the stretched yarns surpasses that of the unstretched ones (77.02).

By Phase III, as the temperature approaches 500 °C and the samples exhibit a relatively rapid weight reduction pace. This is predominantly due to the dehydrogenation reaction as the ladder-like structures transition to graphite-like structures. Following this phase, all TG curves plateau, indicating the formation of a stable structure. The outcomes elucidate that the stretched nanofibrous yarns manifest enhanced thermal stability. Meanwhile, DTG curves for different stretching ratios are shown in Figure 4b. The unstretched yarn loses weight faster than the stretched yarn. This shows that the stretched yarn can form more trapezoidal structures and has higher stability than the unstretched yarn.

TGA can also be used to determine the activation energy associated with random scissions within polymer samples [41]. A higher activation energy indicates greater resistance to random scission under the same conditions. For our samples, we focused on SR 0, SR 6, and SR 8 as representatives of stretched specimens. The samples were subject to different heating rates (5, 10, and 20 K/min) to determine their respective peak temperatures. The Kissinger method can quantify the apparent activation energy without prior understanding of any reaction mechanism. It requires only a series of heating at different rates. Therefore, we employed the Kissinger method to delineate the random scission behavior in these polymers. The results of this analysis are presented in the following table. The Kissinger method [42] is described by the following equation:

(4) $$-\frac{E_{a}}{R}=\frac{d\left[\ln\left(\phi /T^{2}\right)\right]}{d\left(1/T\right)}$$

In this equation, *T* represents the peak temperature corresponding to a specific heating rate ϕ, R stands for the universal gas constant, quantified as 8.314 J mol^−1^ K^−1^, and *E_a_* is the activation energy.

According to the Kissinger method, the activation energies (Table 2) of SR 0, SR 6 and SR 8 are 120.4, 124.8 and 239.2, respectively. The activation energy of SR8 is significantly higher than that of SR0 and SR6, indicating a higher energy barrier and a more difficult reaction for this yarn. It is noteworthy that samples with different stretching ratios show similar behavior, which is attributed to the fact that they are derived from the same type of polyacrylonitrile. As stretching progresses, the molecular chains become oriented. Such oriented chains may be susceptible to certain structural changes upon heating, such as cyclisation of the nitrile groups to form some trapezoidal structures, leading to an increase in the activation energy. At the same time, the increase in crystallinity upon stretching (as evidenced by the XRD results Figure 3) and the stronger intermolecular forces and tighter molecular arrangement of the more crystalline material require greater energy barriers to be overcome during the reaction process, making the stretched yarns more resistant to random breakage. Therefore, SR8 demonstrates greater stability than other yarns. This phenomenon is consistent with the observed increase in breaking strength with increasing stretch ratio (Figure 2).

**Figure 5:**
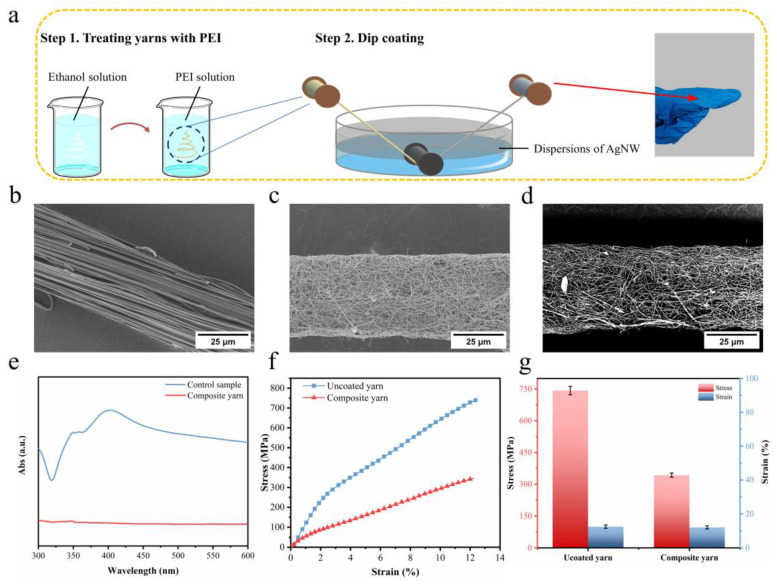
(**a**) Preparation route of the composite yarn. SEM images of uncoated yarn (**b**) and (**c**) composite yarn. (**d**) CBS-SEM image of composite yarn. (**e**) UV/Vis absorption spectrum of the control sample and test sample after washing the composite yarn. (**f**) Stress–strain curves and (**g**) stress and strain of uncoated yarn and composite yarn.

Herein, we demonstrate a conductive composite nanofiber yarn based on our high-strength nanofiber yarn, which uses a straightforward coating method. Figure 5a presents a schematic illustration of the preparation of the conductive composite nanofiber yarn (detailed procedures are described in the Experimental Section). To identify the morphological features of the composite yarn, we examined SEM images of the surface of the nanofiber yarn and composite yarn. From the SEM images, it is evident that the nanofibers are present on the surface of the nanofiber yarn (Figure 5b), whereas no such nanofibers are visible on the surface of the composite yarn (Figure 5c). It was found that the surfaces of the nanofiber yarn in the composite yarn were coated uniformly and randomly with interconnected AgNWs, as confirmed by circular backscatter detector–scanning electron microscopy (CBS-SEM) (Figure 5d).

The stability of the coating was investigated using UV spectra (Figure 5e). The composite yarn was first stirred in deionized water for 30 min. No peaks of AgNWs were observed, indicating that the nanowires were firmly adsorbed on the surface of the yarn and that the coating exhibited excellent stability. The mechanical properties were evaluated using tensile stress versus strain measurements (Figure 5f). The stress of the composite yarn was found to be 343 MPa, which is less than that of the uncoated yarn (Figure 5g). It was postulated that the coating of AgNW on the surface of the composite yarn increased the number of weak knots on the yarn surface, which decreased the stress.

**Figure 6:**
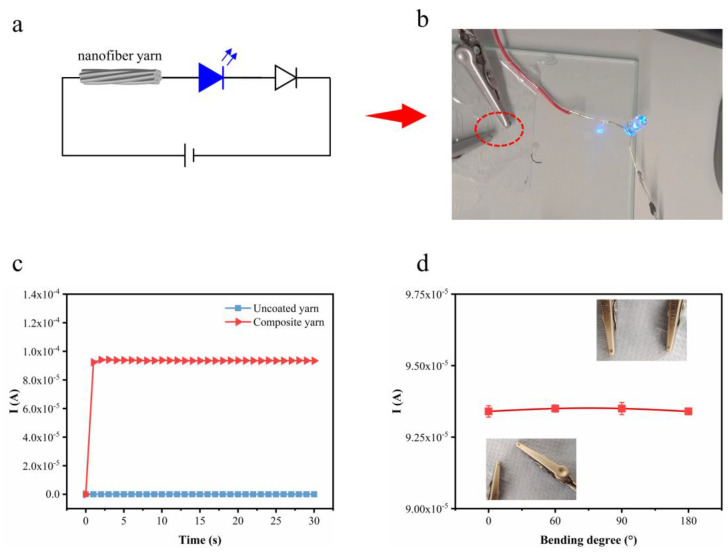
(**a**) Circuit diagram when the yarn is used as an element. (**b**) Photo showing LED light at 3 V using composite yarn as a conductive element. (**c**) Current–time curves of uncoated yarn and composite yarn. (**d**) Current of uncoated yarn and composite yarn versus the bending degree from 0° to 180°. Pictures of the bending process have been inserted.

The electrical performance of the composite yarn was visually demonstrated by illuminating a light-emitting diode (LED) lamp using the composite fiber as circuit wiring (Figure 6a). The LED lamp was observed to be lit at 3 V when AgNW/nanofiber yarn was used as an electrically conductive element (Figure 6b). For the uncoated nanofiber yarn, there was almost no electric current observed over the applied voltage of 3 V, while the composite yarn displayed a noticeable current (Figure 6c). This indicates that AgNWs are physically interconnected on the surfaces of the nanofiber yarn to form a conductive network (Figure 5c). The effect of bending on the electrical properties of the composite nanofiber yarn was investigated. Figure 6d indicates that the current of the composite yarn remained constant even when bent from 0° to 180°. The results of the testing proved the potential application prospects of the composite nanofiber yarn in smart textiles as a robust electrical wiring system.

## 4. Conclusions

In summary, we have developed a novel and facile approach to fabricating PAN nanofiber yarns with high strength (743 MPa) and modulus (1.31 GPa) properties. The yarn was fabricated by using electrospinning and stretching methods. This presented process yields ultrafine electrospun yarns that are sufficiently robust for practical use, due to a combination of thousands of nanofibers with high alignment. Furthermore, benefiting from the dip-coating process, the functionalization of high-strength nanofiber yarns was confirmed to set the stage for its application in implantable flexible/wearable devices. Moreover, our fabrication strategy is versatile and can be extended to diverse materials for the preparation of functionalized nanofiber yarns.

## Figures and Tables

**Table 1:** TGA data of thermal stability for the nanofiber yarns.

Samples	Initial Decomposition Temperature (°C)	Residual Weight at 250 °C (%)	Residual Weight at 350 °C (%)	Residual Weight at 500 °C (%)	Residual Weight at 600 °C (%)
SR 0	286.7	95.43	77.02	49.94	47.23
SR 3	307.2	98.79	80.65	54.20	51.16
SR 6	302.7	98.61	79.77	56.25	53.30
SR 8	303.3	98.14	78.50	52.15	48.86

**Table 2:** *E_a_* determined by Kissinger’s method.

Samples	Rate of Temperature Change (K/min)			*E_a_*
	5	10	20	
SR 0	299.1	320.5	323.9	120.4
SR 6	296.5	316.7	324.8	124.8
SR 8	298.5	307.4	313.9	239.2

## Data Availability

All data generated or analyzed during this study are included in this published article. Any further specific data analysis can be obtained by making a reasonable request to the corresponding author.
